# WHAT IS THE DIFFERENCE BETWEEN OLDER PERSONS WHO CONTINUE HOME CARE AND THOSE WHO DO NOT?

**DOI:** 10.1093/geroni/igad104.2302

**Published:** 2023-12-21

**Authors:** Yuka Sumikawa, Sameh Eltaybani, Chie Fukui, Ayumi Igarashi, Noriko Yamamoto-Mitani

**Affiliations:** The University of Tokyo, Tokyo, Tokyo, Japan; The University of Tokyo, Tokyo, Tokyo, Japan; The University of Tokyo, Tokyo, Tokyo, Japan; The University of Tokyo, Tokyo, Tokyo, Japan; The University of Tokyo, Tokyo, Tokyo, Japan

## Abstract

Enabling older people to remain in and return to their homes after hospitalization or institutionalization (i.e., admission to care institutions, such as nursing homes), if they wish so, is a key principle of aging-in-place worldwide. This prospective cohort study examined the characteristics of older people who returned to their homes after hospitalization or their stay at other care facilities. The study started with 1,450 older persons (≥75 years) receiving home care services at multiple places in Japan. Data were collected at the baseline and 6-month follow-ups. The study was approved by the Research Ethics Committee of the Graduate School of Medicine, The University of Tokyo (No. 2019087NI). At the 6-month follow-up, 256 clients (female: 152 clients, mean age: 85.1 ± 6.1) were hospitalized or institutionalized at least once, of which 54 clients (female: 38 clients, mean age: 83.6± 6.8) returned home afterward. A multivariate logistic regression showed that females (odds ratio [95% confidence interval]: 2.01 [1.06 to 4.09]), those with more care assistance (1.27 [1.03 to 1.55]) were more likely to return to their home, whereas older clients had a lower likelihood (0. 93 [0.88 to 0.98]). Based on this finding that younger females were more likely to return home if hospitalized or institutionalized, older males may have different needs than females, influencing the type and level of support they require. Further research might identify the support needed for older males with less care assistance.

